# Cigarette-Smoke-Induced Dysregulation of MicroRNA Expression and Its Role in Lung Carcinogenesis

**DOI:** 10.1155/2012/791234

**Published:** 2011-12-01

**Authors:** Rebecca Russ, Frank J. Slack

**Affiliations:** Department of Molecular, Cellular and Developmental Biology, Yale University, P.O. Box 208103, New Haven, CT 06520, USA

## Abstract

Dysregulation of microRNAs (miRNAs), particularly their downregulation, has been widely shown to be associated with the development of lung cancer. Downregulation of miRNAs leads to the overactivation of their oncogene targets, while upregulation of some miRNAs leads to inhibition of important tumor suppressors. Research has implicated cigarette smoke in miRNA dysregulation, leading to carcinogenesis. Cigarette smoke may lead to genetic or epigenetic damage to miRNAs, many of which map to fragile sites and some of which contain single nucleotide polymorphisms. Cigarette smoke may also cause dysregulation by affecting regulatory mechanisms controlling miRNA expression. Researchers have shown a correlation between smoke-exposure-induced dysregulation of miRNAs and age. Furthermore, dysregulation seems to be associated with intensity and duration of smoke exposure and duration of cessation. Longer exposure at a threshold level is needed for irreversibility of changes in expression. Better understanding of miRNA dysregulation may allow for improved biomonitoring and treatment regimens for lung cancer.

## 1. Introduction

In the United States, lung cancer is the second most common cancer to occur in men and women [1], yet with a five-year survival rate of only 15%, lung cancer represents the leading cause of cancer-related deaths [1, 2]. This poor survival is largely a product of lack of early screenable biomarkers, which leads to detection of the cancer at an advanced and typically untreatable stage [2]. Furthermore, histologic classification of lung cancer is limited, given the genotypic heterogeneity among cells of a similar type [3]. Such heterogeneity causes varied responses to treatment and limits the efficacy of a single or generalized therapy. Previous research has analyzed gene expression signatures to elucidate the heterogeneity of lung cancers, allowing for more accurate diagnosis and prognosis and permitting creation of more targeted therapies [3, 4]. Nevertheless, as mRNAs may be regulated posttranscriptionally, only determining mRNA expression may not fully portray the biological mechanisms involved in cancer initiation and progression [2]. Computer models have predicted that miRNAs, which posttranscriptionally suppresses translation of mRNA, accounts for 1/3 of total regulation of the genome [5]. Dysregulation of miRNA, thus, can have an important consequence in the dysregulation of genes, and miRNA analysis can provide a more comprehensive understanding of lung cancer pathogenesis. Moreover, research has found that exposure to cigarette smoke can lead to alterations in miRNAs, establishing a pivotal connection between cigarette smoke and carcinogenesis [6]. Elucidating the dynamics and carcinogenic mechanisms behind the cigarette smoke-induced dysregulation of miRNAs will contribute to the development of more effective lung cancer diagnosis, chemoprevention, and therapy.

## 2. miRNA Processing

About 1000 human miRNAs have been identified (MirBase), and while much about their function and specific biological targets is unknown, researchers are clarifying the general mechanisms of miRNA expression (transcription and processing) and suppression of mRNA [2]. Processing begins with a large primary transcript, pri-miRNA, of >100 nucleotides in length, transcribed by RNA polymerase II. The newly transcribed pri-miRNA is bound to double-stranded RNA binding domain protein within the nucleus. The pri-miRNA then binds to DiGeorge syndrome critical region gene 8 protein (DGCR8) and an RNase III endonuclease (Drosha). This Microprocessor complex cleaves the pri-miRNA to a 70 nucleotide imperfect stem loop precursor called pre-miRNA. Exportin 5 and Ran GTPase then transport pre-miRNA from the nucleus to the cytoplasm where it forms a second processing complex containing the RNase III endonuclease Dicer and the transactivating response RNA binding protein (TRBP). This complex cuts the stem loop pre-miRNA into a double-stranded miRNA of about 22 nucleotides in length. The strands are separated and AGO proteins help incorporate the antisense strand into the RISC complex. Once in the RISC complex, the AGO proteins bind in the 3′ end of mRNA, stabilizing the interaction between the miRNA and mRNA. The miRNA targets the specific mRNAs through complementarity between the 5′ end of the miRNA and the 3′ UTR of the mRNA. MiRNAs that have perfect complementarity with the duplexed mRNA initiate the RNA-mediated interference pathway, through which the target mRNA is cleaved by the AGO proteins and then degraded [2, 7]. Imperfectly binding miRNAs prevent translation of the target mRNAs and causes mRNA instability [7].

## 3. Dysregulation of miRNA

Several studies have recently shown that exposure to cigarette smoke in both humans and rats leads to global alterations in miRNA expression.  Table 1 provides a summary of important miRNAs that are significantly dysregulated by cigarette smoke and are implicated in carcinogenesis. After exposing rats to environmental cigarette smoke for 28 days and examining miRNA expression in normal lung cells, the Izzotti group found that of the 484 miRNAs analyzed, 126 miRNAs (26%) were downregulated twofold. Of the 484 miRNAs analyzed, only 7 miRNAs (1.4%) were upregulated twofold. Moreover, 24 miRNAs were downregulated threefold with a statistically significant variation between the rats exposed to environmental smoke and those not exposed [8]. Cigarette-smoke-induced dysregulation of miRNA has been demonstrated in humans as well. Examining the bronchial airway epithelium of current smokers and never smokers, Schembri et al. found 28 miRNAs to be significantly differentially expressed. Of the 28 dysregulated miRNAs, 23 were downregulated [9]. MiR-30a was commonly downregulated in both humans and rats [8, 9]. Among studies analyzing cigarette-smoke-dysregulated miRNA, the downregulation of miRNA seems to be more common than upregulation [8–10]. With their pivotal regulation of mRNAs, carcinogens like cigarette smoke that cause alterations in miRNA levels can cause miRNAs to act as oncogenes (or fail to act as tumor suppressors), thereby rendering smokers susceptible to the development of lung cancer [10].

## 4. Mechanisms of Cigarette-Smoke-Induced miRNA Dysregulation

**Figure 1:**
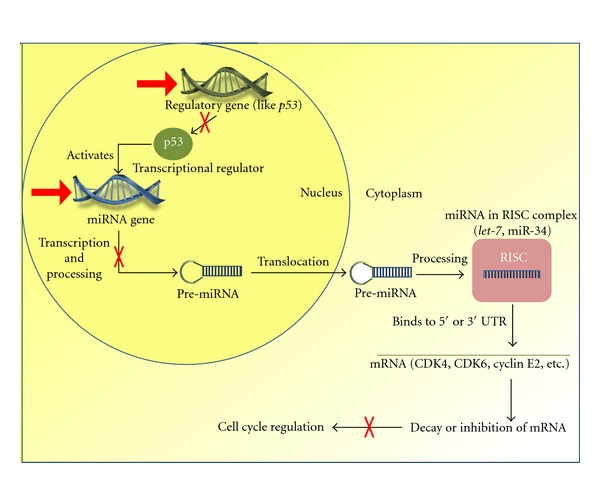
Simplified overview of the mechanisms by which cigarette smoke induces dysregulation of miRNA (red arrows) and subsequent carcinogenesis. CS can lead to genetic mutations in miRNA transcriptional regulators, like P53. Dysregulation of P53, an activator of miRNA transcription, leads to downregulation of miRNA, like miR-34. CS can also lead to epigenetic or genetic mutations in the miRNA gene itself, leading to dysregulation of transcription. Both downregulation and upregulation of miRNA can allow for cell proliferation if the miRNA is a tumor suppressor or oncogene, respectively. The red arrows indicate direct action of CS on miRNA production. The red “X's” indicate inhibition of a normal process (indicated by black arrows).

The exact mechanism by which cigarette smoke causes this dangerous dysregulation remains unknown; nevertheless, recent studies are beginning to offer theories elucidating the mechanism.  Figure 1 depicts basic mechanisms by which cigarette smoke may induce dysregulation of miRNA and may lead to cell cycle dysregulation. Tobacco smoke contains free radicals and oxidative compounds which are highly mutagenic [8]. The tar phase of smoke contains a quinone and hydroquinone complex within the tarry matrix. This complex is highly oxidative, producing hydrogen peroxide and hydroxyl radicals. The gas phase of cigarette smoke is equally harmful, containing small oxygen and carbon-centered radicals. Such radicals have been demonstrated to react with DNA in vitro [11]. For example, aqueous cigarette tar (ACT) solutions have been shown to react with DNA nucleotides to create adducts like 8-hydroxy-2′ deoxyguanosine, which has been used as a biomarker for carcinogenesis [12].

 In addition to the high toxicity and mutagenicity of cigarette smoke, the genes for miRNAs often lie in particularly mutable parts of the genome. After mapping 186 miRNA genes and comparing their location to sites previously reported as genetically mutated, Calin et al. found that the miRNAs are often located in “fragile sites” of the genome [13]. Esquela-Kerscher and Slack noted that many human homologues of the *let-7* family (see below) map to such fragile sites [7]. Furthermore, as cigarette smoke seems to act through continued exposure rather than a single initiating event, some miRNA alterations may also be a product of disruption in epigenetics rather than initiating mutational events [8].

In addition to being located at fragile sites in the genome, many miRNAs are characterized by single polymorphisms rendering them even more susceptible to genetic damage by cigarette smoke. The Izzotti et al. experiment found five cigarette smoke downregulated miRNAs with single nucleotide polymorphisms [8]. One of these miRNAs is miR-125. In addition to the family members mapping to 11q23-q24 and 21q11.1, fragile sites commonly deleted in lung cancer patients, *mir-125a* has a G/U polymorphism at nucleotide 8 [8, 13, 14]. The uracil polymorphism inhibits processing of the primary to the precursor miRNA. With a single nucleotide altering its processing, this miRNA is more susceptible to genetic damage and subsequent downregulation by carcinogens like cigarette smoke [8, 14]. miR-125 normally suppresses ERBB7, the oncogenic coding sequence for EGFR, a growth factor receptor often overexpressed in carcinomas [14, 15]. Therefore, if genetic damage inhibits processing and downregulates miR-125, it may lose its functionality as a tumor suppressor of EGFR. Given that some miRNAs have easily mutable single nucleotide polymorphisms which can singly determine their functionality, alterations in miRNA levels may be an early event in cigarette-smoke-induced carcinogenesis [8]. In addition to their importance in early carcinogenesis, single nucleotide polymorphisms in miRNA genes may account for variations in carcinogenic susceptibility among smokers [16].

Cigarette smoke may also cause the dysregulation of miRNAs by disrupting miRNA regulatory mechanisms, like the P53 pathway. The tumor suppressor P53 is an important regulatory protein that can induce cell cycle arrest, cell growth, apoptosis, and angiogenesis [17]. Over half of all lung cancers have a mutation in P53, and the frequency of these mutations seems to be dependent on smoking status and number of cigarettes smoked [18–20]. Smoke seems to leave a unique “molecular signature” on the P53 gene in smoke-exposed lung tumors. Smoke exposure is often correlated with an increase in G to T transversions in the gene, possibly caused by a reaction with polycyclic aromatic hydrocarbons in tobacco smoke [21]. Normally, P53 directly transactivates and promotes the transcription of *mir-34a–c*, which in turn induces cell cycle arrest by targeting proteins such as CDK4, CDK6, cyclin E2, and E2F3 [17, 21]. However, once mutated, P53 may fail to efficiently induce miR-34 expression, leading to miR-34′s downregulation. Indeed, as Izzotti et al. showed, miR-34 is, in fact, downregulated in the presence of environmental cigarette smoke. (In this experiment, it was specifically downregulated a striking 3.6-fold [8].) Nevertheless, this dysregulation may not entirely depend on the loss of P53, as miR-34 downregulation has been shown to independently diminish the efficiency of the P53-dependent apoptosis, suggesting its independent and crucial role in the p53 pathway and in tumorogenesis [22, 23].

One family of miRNA, the *let-7* family, has been closely studied for its role in the development of lung cancer and for its potential use in therapy; recently, exposure to cigarette smoke has been implicated in the downregulation of *let-7*, offering one possible mechanism for carcinogenesis. *let-7* and its family are highly conserved across animal species [8]. Humans have ten mature *let-7* sequences, denoted by the letters “a” through “k,” and 13 precursor *let-7* species [24]. One of the genes that *let-7* seems to directly regulate is the oncogene *RAS*. Ras proteins are GTPase proteins associated with the plasma membrane. The proteins promote cellular growth and differentiation. Approximately 15–30% of all human tumors have mutations in *RAS* that stimulate Ras overexpression and cause oncogenic transformation of the cell [7]. By targeting LCSs (*let-7* complementary sites) in the 3′ UTR of *RAS*, *let-7* post-transcriptionally downregulates the expression of the gene. In lung tumors, *let-7* seems to be downregulated compared to normal tissue. As a response to the decreased miRNA repression, Ras is upregulated [25]. Other direct targets of *let-7* include cyclins like A2, which signals G1-S and G2-M transitions, and CDKs like CDK6. CDK6 interacts with D cyclins to phosphorylate RB1, ultimately promoting the G1 phase of the cell cycle [26]. The activator of S-phase kinase (ASK), necessary for DNA replication in the G1 to S transition, is also under the control of the *let-7* family. In total, Johnson et al. found that changes in *let-7* levels significantly affected close to 200 genes in both liver and lung cells. Thus, the *let-7* family seems to be a “master regulator” of cell proliferation, directly or indirectly controlling the expression of many cell-cycle genes. In the same experiment, the addition of synthetic *let-7* caused a cell cycle deficiency, in which the percentage of cells stuck in the G0-G1 phase increased. At normal levels, it seems that *let-7* helps delay the G1-S transition [27]. The downregulation of this master regulator seems to be a typical and initiating event in the development of lung cancer, promoting cell progression through the G1-S transition [2, 27, 28].

The mechanism of *let-7* carcinogenesis is further understood in the context of smoke exposure. Several experiments have found significant downregulation of *let-7* and upregulation of its targets after exposure to cigarette smoke [8, 29, 30]. Izzotti et al. found a large 4.6-fold decrease in *let-7c* in lung tissue of rats after only four weeks of exposure to environmental cigarette smoke [8]. Such data supports the group's previous proteome data that *let-7*-targeted cyclins and CDKs were upregulated in the same tissue [30].

In addition to tumor suppressors like *let-7*, miRNAs may serve as oncogenes; subsequently, their cigarette-smoke-induced upregulation can contribute to carcinogenesis [5]. In the Izzotti et al.'s experiment, after rats were exposed to environmental cigarette smoke for four weeks, the expression of 484 miRNAs in the lung tissue was analyzed. Of these miRNAs, while 126 (26%) showed a twofold downregulation, only 7 miRNAs (1.4%) showed an upregulation of twofold compared to sham-air exposed rats. Similarly, compared with sham rats, while 26 miRNAs were significantly downregulated threefold, only 1 was significantly upregulated. This miRNA, miR-294, was upregulated a significant 10.7-fold. In normal expression, miR-294 silences transcriptional repressor genes like zinc finger protein 697 and AT-rich interactive domain 4A. However, when upregulated, miR-294 promotes a global increase in transcription, potentially becoming oncogenic [8].

## 5. Age Association, Dose Responsiveness, and Persistence of Dysregulation

Regardless of whether miRNAs serve as oncogenes or tumor suppressors, their cigarette-smoke-induced dysregulation seems to vary with age. In another experiment by the Izzotti group, newborn mice were exposed to environmental cigarette smoke for five weeks, corresponding to the weanling period. The miRNA expression in their lung tissue was subsequently compared to postnatal untreated mice, adult mice exposed to five weeks of cigarette smoke, and newborn and adult mice exposed to sham air for five weeks. In mice not exposed to smoke, the largest variations in miRNA occurred during the weanling period. This seems congruent with that fact that there is a large amount of oxidative stress in the lung of a newborn during the transition from “the maternal-mediated respiration of the fetus to the autonomous pulmonary respiration of the newborn.” All 11 miRNAs that were significantly altered from birth to the end of the weanling period are involved in early life processes. Several were involved in embryological development and morphological changes, “reflecting the fact that development and maturation of the lung are completed after birth” [31]. During this transition to pulmonary respiration, oxidative stress on the newborn lung induces many genomic and postgenomic alterations, including a significant increase in bulky DNA adducts and oxidatively damaged DNA [32]. Cells may respond to such genomic damages by increasing expression of stress-response genes through downregulation of miRNAs. In the experiment, 7 downregulated miRNAs in the postweanlings were involved in proliferation and differentiation, processes contributing to stress-response during lung development [31].

In mice exposed to environmental cigarette smoke, both the adult and postweanling mice revealed predominantly downregulated miRNAs. Since many of the downregulated miRNAs allow for the overactivation of oncogenes, miRNA variation in both postweanlings and adults can contribute to miRNA-mediated carcinogenesis. Among the miRNAs downregulated were *let-7b, *miR-26a, miR-30c, miR-124a, miR-125a, miR-192, and miR-431, the downregulation of which promotes activation of oncogenes and growth factors like Ras, ERBB2, EGF, and TGF. Tumor suppressors like *let-7* and effectors of the p53 pathway, miR-34b and miR-140, were also downregulated, revealing their possible role in smoke-mediated carcinogenesis.

Nevertheless, the postweanling, smoke-exposed mice had a greater downregulation than the adult, smoke-exposed mice. MiR-26a and miR-140 had the greatest difference in downregulation, between postweanling females and to adult females. The downregulation of miR-26a leads to the overexpression of TGF growth factor, whereas the downregulation of miR-140 suppresses the p53 pathway [32]. These two pathways offer possible mechanisms of carcinogenesis in mice exposed early in life. Mice exposed to smoke early in life do, indeed, have a high incidence and development of lung tumors. In the study by Balansky et al., newborn mice exposed to mainstream cigarette smoke (MCS) quickly developed a large yield of lung microadenomas and adenomas in addition to bronchial and alveolar hyperplasia. The untreated control mice had no tumors. The authors posit that the mice have a “striking” “susceptibility…to the carcinogenicity of MCS, when exposure starts few hours after birth” [33]. The great downregulation of miRNAs in ECS-exposed weanling mice may be correlated with this “striking” tumorogenesis.

Compared with adult mice, the high susceptibility of newborn mice to carcinogenesis may be a product of the synchronization of the smoke-exposure with their vulnerable lung maturation process. During this maturation process, natural oxidative damage of the DNA occurs in addition to smoke-caused oxidative damage. As newborn organs have an increased rate of proliferation, there is higher risk for clonal expansion of mutated cells [33, 34]. The downregulation of miRNAs in neonatal mice may allow such hyperplasia to go unchecked [32, 33]. Finally, neonatal mice have “hypothetically, an increased probability for the involvement of stem cells, which have been shown to have an increased susceptibility to genotoxic carcinogens” [35].

Though many studies have profiled the smoke-induce differential expression of miRNA, few have evaluated the dynamics and persistence of such alterations. The Izzotti group has begun initial research into the dose-responsiveness and persistence of smoke-induced miRNA alterations. In the Izzotti experiment, neonatal mice were exposed to sham air and three different doses of smoke: 119 mg/m^3^, 292 mg/m^3^, and 438 mg/m^3^ total particulate matter (TPM) for four weeks. Lung tissue exposed to each of the doses was analyzed for bulky DNA adduct and 8-oxodGuo levels, as well as differential expression of miRNA. In the 119 and 292 mg/m^3^ TPM-dosed lung tissue, bulky DNA adduct and 8-oxodGuo levels significantly increased. However, miRNA expression was not altered. Only at the highest dose of 438 mg/m^3^ TPM did the miRNA expression change significantly. This dose responsiveness, thus, indicates that a threshold dose of cigarette smoke exposure is required to alter miRNA expression and induce miRNA-mediated carcinogenesis [36].

**Figure 2:**
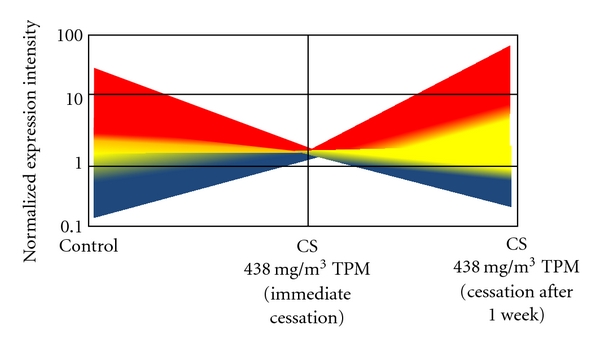
Persistence of miRNA in lung tissue: cartoon of a graph adapted from [36] of the intensity of expression of 697 miRNAs in the lung tissue of untreated mice or mice exposed to 438 mg/m^3^ TPM of cigarette smoke for 4 weeks. The mice were sacrificed either immediately (1 day) or 1 week after cessation. The blue to red gradient represents the least to most intense miRNA expressions, respectively. The graph reveals that most of the changes in miRNA expression were reversed after 1 week of cessation.

In the second phase of the experiment, the Izzotti group analyzed the persistence of miRNA expression alterations. The group compared DNA damage and miRNA expression in mice exposed to 438 mg/m^3^ TPM that were sacrificed immediately, after one week of cessation, and after four weeks of cessation. After one week of cessation, miRNA downregulation was decreased (Figure 2). Thus, the team concluded that four weeks of smoke exposure, even at the highest dose, was too short of a time to induce irreversible changes in miRNA expression. In light of the reversible downregulation of miRNA, 4 weeks is too short to induce miRNA changes that could be involved in smoke-induced carcinogenesis [36]. The longer the exposure to cigarette smoke, the longer the alterations in lung miRNA expression persist and are potentially pathological. Mice exposed to mainstream cigarette smoke for four months experienced alterations in miRNA which lasted for three months [14].

 The reversibility of cigarette smoke-induced miRNA changes in the lung may be a manifestation of the adaptive response mechanisms of the lung cells. Upon less intense and shorter exposure to cigarette smoke, cells down-regulate many miRNAs to “activate adaptive and protective functions aimed at defending the respiratory tract from the adverse effects” of the smoke. Indeed, many of the downregulated miRNAs in cigarette smoke exposure studies are involved in this response to mediate the toxic effects of smoke. Such miRNAs include miR-122a, miR-431, and miR-99b [31]. MiR-122 downregulation increases heme oxygenase-1 (HO-1) activity, which has been found to act as an antioxidant in both the liver and the lungs of cigarette smoke-exposed mice [37, 38] MiR-431 is involved in protein repair, while miR-99b induces apoptosis of smoke-damaged cells [31]. One of the most significantly downregulated miRNAs is miR-30a [8, 36]. The downregulation of this miRNA through smoke-exposure may allow for an increased release of NF-*κ*B inhibitors, allowing for NF-*κ*B's nuclear translocation and activation. Thus, downregulation of miR-30a activates the NF-*κ*B pathway and stimulates an inflammatory response [8, 30, 36]. Other downregulated miRNAs, like *let-7a*, miR-30b, miR-30c, miR-124a, miR-219, and miR-376, increase cell proliferation. Cell proliferation, known to increase in the lungs upon exposure to smoke, replenishes the smoke damaged lung tissue [38, 39].

Although the miRNAs studied had reversible alterations in expression that contributed to the adaptive response, some miRNAs took a longer time to recover to normal levels; surprisingly, those that persisted for longer seemed to be miRNAs previously implicated in carcinogenesis. Many of the miRNAs that remained downregulated after one week of cessation were involved in the P53 pathway. MiR-34b, as mentioned before, is a main effector of the P53 tumor suppressor pathway. MiR-345, miR-421, and miR-450, also persisting for at least one week of cessation, are normally involved in the inhibition of the RAS oncogene pathway. The longer oncogenic miRNA alterations like these persist, the more likely they will contribute to the dysregulation of the lung cells and the initiation of cancer [36].

## 6. Conclusions

The Izzotti study clarifying dose-responsiveness and persistence of miRNA alterations shed light on the timeline of cigarette-smoke-induced carcinogenesis. Cigarette smoke causes irreversible miRNA expression changes only if animals are exposed for a sufficient period of time at high doses surpassing a threshold [36]. More in depth research is needed as to what doses and length of time are needed for such irreversibility in both rodents and humans. However, such conclusions about the dynamics of cigarette smoke regulated miRNA expression have implications for public health. The International Agency for Research on Cancer (IARC) cites that “in smokers, the most important parameter of smoking that affects lung cancer risk is the duration of regular smoking, although the risk also increases with the number of cigarettes smoked per day.” This statement supports the idea that duration of smoke exposure seems correlated with reversibility of carcinogenic miRNA changes. Duration seems more important than intensity, as it seems there is a threshold level of smoke below which carcinogenic changes may be negligible. Furthermore, similar to the Izzotti et al. study of persistence [36], smoker cessation for at least one to four years is correlated with a significantly lower risk of lung cancer. The relative risk for lung cancer decreases further with increased cessation time. Though more research is needed, reversible changes in miRNA expression may be associated with this mutability of risk [40].

### 6.1. miRNA as a Biomonitoring Tool

With the recent research into miRNA expression in smoke exposed lung tissue, miRNA expression profiling may soon become an important tool for biomonitoring the risk and development of lung cancer. MiRNA expression profiles have proven to have higher sensitivity to cigarette smoke (larger changes in quantity expressed) than other proteomic and transcriptomic profiles. For example, of 484 miRNAs profiled, 126 (26.0%) had at least twofold downregulation. In the same tissue, cigarette smoke induced a twofold upregulation of only 2.9% of genes and 9.7% of proteins [8]. This increased sensitivity could lead to more detailed monitoring of activated and inhibited pathways in cancer progression, allowing for the elaboration of carcinogenic mechanisms. More sensitivity could also allow for improved estimation of cancer risk.

New techniques in miRNA monitoring could also lead to better cancer diagnosis. In the past, miRNA microarrays have been used to examine the tissue-specific expression profiles (called signatures) of miRNA genes [10, 41]. Lu et al., however, developed a new and more accurate technique to study miRNA signatures: the bead-based flow cytometry technique. This technique involves carboxylating oligonucleotide probes to 5-micron polystyrene beads. Each bead was filled with two fluorescent dyes, creating variations of colors and allowing for one color to represent one miRNA being examined. After hybridizing the miRNAs to the probe, a flow cytometer is used to detect color and intensity of the beads, thus, quantifying miRNA expression. This method has proven a highly accurate way to diagnose and stage tumors: the profiles can be used to differentiate normal and tumor tissues, classify cellular differentiation, and determine developmental origin. Compared to mRNA tumor profiling, with relatively few miRNAs analyzed, miRNA profiling accurately classified tumors that were poorly differentiated [41].

Moreover, miRNA signatures can predict an accurate prognosis of the tumor [7]. In a recent study, neonatal mice were exposed to 723 mg/m^3^ TPM of mainstream cigarette smoke for 120 days and were taken off treatment for three months. Some mice had preexisting pulmonary adenomas, carcinomas, or pneumonias, and some of these were treated with either the N-acetyl-l-cysteine (NAC), 5,6-benzoflavone, or phenethyl isothiocyanate (PEITC) chemopreventatives. The miRNA expression profiles were compared for the different cancers, and it was found that each type of cancer had specific dysregulation of miRNA that was significantly predictive of the diagnosis. For example, pulmonary adenoma had a predictive downregulation in miR-10b, miR-30c, and miR-138. miR-10b is overexpressed in adenomas in proportion to the aggressiveness of the tumor. Its downregulation in this study supports that the tumor's prognosis was benign [14].

### 6.2. Chemoprevention of miRNA Dysregulation

Profiling miRNAs can also predict the efficacy of chemoprevention [7]. miR-30c and miR-138 are both implicated in chemotherapy resistance and coordinated by the expression of the MDR-1 gene. Remarkably, in mice treated with NAC or PEITC, both miRNAs are upregulated, potentially diminishing chemoresistance. Such upregulation reveals the efficacy of both treatments. Bronchoalveolar carcinoma had a predictive and striking downregulation of *let-7* by 6.1-fold. This downregulation was attenuated through chemoprevention with PEITC [14]. In a different experiment, 484 miRNAs from lung tissue of cigarette-exposed rats were monitored for their response to the chemopreventative agents NAC, oltipraz (OPZ), indole-3-carbinol (I3C), PEITC, and 5,6 benzoflavone (BF). None of the chemoagents had a significant effect on the baseline miRNA levels of the untreated group of mice, signifying the agents' safety. At the same time, all of these agents modulate miRNA levels in the smoke-exposed rats. Rats treated with a combination of OPZ and PEITC saw a near normalization of several miRNAs like *let-7*, pre-miR-191, and pre-miR-222. This upregulation suggests a reduction in proliferation. By allowing researchers to predict the efficacy of chemopreventative agents, researchers can screen agents and continue to develop those that seem highly effective. In the long-run, this increases cost and time efficiency of chemopreventative research. Many of the miRNAs targeted by the chemopreventative agents in this experiment (*let-7a, *miR-125b, miR-140 s, and pre-miR-146) are polymorphic in humans. It may be possible to use miRNA profiling to understand not only individual susceptibility to smoke-induced carcinogenesis, but also how these polymorphisms and chemopreventative agents interact to regulate the expression of miRNA. It, thus, might be possible to predict how specific individuals might react to certain chemopreventative agents. In the future, miRNA profiling may allow for personalized and highly effective cancer prevention [42].

### 6.3. miRNA as a Therapeutic Agent

MiRNA profiling in cigarette-smoke-exposed tissue and lung cancer tissues can and has contributed to the development of novel therapies using miRNA itself. The downregulation of tumor suppressor miRNAs, like *let-7*, is critical to lung carcinogenesis. Exogenous delivery of miRNAs like *let-7* to patients at risk of cancer may prevent its development. Slack and Weidhaas hypothesize that patients with lung tumors containing activating *KRAS* mutations would also be ideal candidates for such a therapy; exogenous *let-7* would add another block to the development of the cancer. Slack and Weidhaas suggest two possible methods of miRNA delivery, including viral gene therapy or modified miRNA [28]. Liposomal delivery of miRNA might also be useful. All of these approaches would require the delivery of hairpin and flanking sequences of pre-miRNA. Through stimulation of tissue-specific promoters, such therapy would rely on endogenous regulation to process and promote miRNA-mediated repression of genes. The Slack group has found success with *let-7* therapy; its delivery not only seems to prevent the development of premalignant lung tumors, but also shrinks those *RAS*-activated tumors [7]. In overexpressed miRNAs, antisense oligonucleotides for the dysregulated miRNAs can be synthetically created. These antisense molecules, called antimiRNA oligonucleotides (AMOs), work by targeting exactly complementary sequences in the overexpressed miRNA. Conjugated with cholesterol for delivery, these AMOs have successfully inhibited the oncogenic miRNA activity in mice, and they seem to be much more stable and less toxic than other cancer treatments [43, 44]. While it is unlikely that miRNA therapy alone will be sufficiently effective, the therapy may effectively treat cancer when combined with other treatments. For example, tumors with an overactive EGFR-Ras pathway are very common but are often untreatable due to the chemoresistance of the cells [28]. Use of farnesylation inhibitors to inhibit RAS membrane targeting and inhibition of EGFR have been ineffective [45, 46]. The inefficacy of such treatments is probably a result of the lack of specificity of the inhibitors for the Ras pathway [47]. With the specific complementarity of miRNA, exogenous delivery of *let-7* could inhibit RAS membrane targeting, “bypassing the radioprotective effects of the EGFR-RAS pathway [28]”.

### 6.4. Final Thoughts

Dysregulation of miRNAs, and particularly their downregulation, has been widely shown to be associated with the development of lung cancer. Recently, research has implicated cigarette smoke in the induction of such miRNA dysregulation, leading to carcinogenesis. Scientists have found that cigarette smoke may lead to genetic or epigenetic damage of miRNA genes, many of which are at fragile sites and many of which have vulnerable single nucleotide polymorphisms. Cigarette smoke may also cause dysregulation by affecting regulatory mechanisms like the p53 tumor suppressor. The downregulation of miRNAs like the *let-7* family leads to the overactivation of their oncogene targets. Upregulation in a few miRNAs leads to inhibition of important tumor suppressors. Recently, researchers have shown a correlation between smoke exposure-induced dysregulation of miRNAs and age; newborn rodents seem to be the most vulnerable to both miRNA downregulation and tumorogenesis. This is perhaps due to the significant natural oxidative stress and the vulnerability of their newly developing lungs. Furthermore, the dysregulation seems to be associated with intensity of smoke exposure, duration of exposure, and duration of cessation. Dose-responsiveness studies of the dysregulation suggest a threshold of exposure is necessary for significant changes in miRNA expression. Short-term exposure leads to reversible changes that seem associated with an adaptive response mechanism. Longer exposure is needed for irreversibility of changes in expression. The exact mechanisms and dynamics of cigarette-smoke-induced dysregulation are largely unknown. Nevertheless, with a growing understanding, researchers may be able to elucidate an important player in cigarette-smoke-caused lung carcinogenesis. With better understanding of miRNA, researchers may have the chance to improve biomonitoring and treatment technologies. Defining the role of miRNA in cigarette-smoke carcinogenesis could lead to improved diagnosis, prognosis, and treatment of lung cancer.

## Figures and Tables

**Table 1:** Important miRNAs significantly dysregulated by cigarette smoke and implicated in carcinogenesis [8, 14, 32, 36].

miRNA	Targets	Biological effects of dysregulation	Comments on dysregulation
Downregulated			

*let-7a, b, c, f*	Ras, CDK6, cyclin A2	Ras oncogene activation, cell proliferation, angiogenesis	Normally, *let-7* is a master regulator of cell proliferation
*mir-26a*	TGF	TGF overexpression, angiogenesis	Greater downregulation in postweanling female mice
*mir-30a*	EFG pathway, NF-*κ*B inhibitors, *CDC40 *	Cell cycle progression, cell adhesion, protein repair, EFG activation, stress response (NF-*κ*B activation)	Downregulated in both humans and rats
*mir-30c*	EFG pathway, NF-*κ*B inhibitors, *CDC41 *	Chemoresistance, cell cycle progression, cell adhesion, protein repair, EFG activation, stress response (NF-*κ*B activation)	Downregulation leads to chemoresistance mediated through MDR-1
*mir-34 a–c*	CDK4, CDK6, cyclin E2, and E2F3	Cell cycle arrest inhibited	Mutations in p53 can lead to its downregulation
*mir-122a*	heme-oxygenase 1	Increased antioxidant activity in liver and lungs	Involved in stress response
*mir-125a*	ERBB7 (gene for EGFR)	ERBB7 oncogene activation	Maps to fragile site with G/U polymorphism
*mir-140*	p53 pathway	Cell cycle arrest inhibited	Greater downregulated in postweanling female mice
*mir-345*	Ras pathway	Ras oncogene activation, cell proliferation	Downregulation is persistent for at least one week after cessation

Upregulated			

*mir-294*	Zinc finger protein 697, AT-rich interactive domain 4A	Increase in transcription	Upregulated 10.4-fold in Izzotti et al. [8].
